# Subxiphoid uniportal thoracoscopic pulmonary segmentectomy for stage I non‐small cell lung cancer: Feasibility, quality of life and financial worthiness

**DOI:** 10.1111/1759-7714.13392

**Published:** 2020-03-28

**Authors:** Amr Abdellateef, Xiaoyu Ma, Zhigang Chen, Liang Wu, Jianqiao Cai, Lei Jiang

**Affiliations:** ^1^ Department of Cardiothoracic Surgery, Mansoura University Hospital, Mansoura School of Medicine Mansoura University Mansoura Egypt; ^2^ Department of Thoracic Surgery Shanghai Pulmonary Hospital, Tongji University School of Medicine Shanghai China; ^3^ Thoracic Surgery Department Second Hospital of Hebei Medical University Shijiazhuang China; ^4^ Department of Anesthesiology, Shanghai Pulmonary Hospital Tongji University School of Medicine Shanghai China

**Keywords:** Cost, segmentectomy, subxiphoid, thoracoscopic, uniportal

## Abstract

**Background:**

Subxiphoid uniportal video‐assisted thoracoscopic surgery (SVATS) is more technically challenging than intercostal uniportal video‐assisted thoracoscopic surgery (UVATS), especially in more complex procedures such as segmentectomy. We therefore aimed to investigate the worthiness of undertaking the more demanding subxiphoid approach in patients who had undergone anatomical segmentectomy for stage IA non‐small cell lung cancer (NSCLC).

**Methods:**

A total of 491 patients were included in our study who had undergone anatomical segmentectomy for stage IA non‐small cell lung cancer from September 2014 to April 2018. They were divided into two groups; 278 patients in the UVATS group and 213 patients in the SVATS group. Different perioperative variables, postoperative pain, quality of life and cost were analyzed and compared between both groups.

**Results:**

The SVATS group showed a significantly longer operative time (*P* = 0.007) and more operative blood loss than the intercostal group (*P* = 0.004). There was no significant difference between both groups regarding postoperative drainage, duration of chest tube, postoperative hospital stay, operative conversion or postoperative complications. The SVATS group showed a significantly lower pain score postoperatively (*P* < 0.001). In addition, the SVATS group showed a significantly better postoperative quality of life score along the first postoperative year (*P* < 0.001). UVATS segmentectomy appeared to be significantly cheaper than SVATS segmentectomy (*P* < 0.001).

**Conclusions:**

SVATS segmentectomy for stage IA lung cancer is a safe procedure that is worth proceeding with as it is associated with better postoperative pain and better quality of life in the first postoperative year. Further studies are recommended to evaluate the actual cost‐effectiveness of SVATS segmentectomy.

**Key points:**

• **Significant findings of the study**

Subxiphoid uniportal approach for pulmonary segmentectomy is safe and feasible approach. It has better postoperative pain and better quality of life than the uniportal intercostal approach; however, it is more expensive.

**• What this study adds**

Subxiphoid uniportal approach for pulmonary segmentectomy gives a better quality of life in Chinese patients than the intercostal approach; however, it is more expensive.

## Introduction

Subxiphoid uniportal video‐assisted thoracoscopic surgery (SVATS) has been introduced as safe and feasible alternative approach for intercostal uniportal video‐assisted thoracoscopic surgery (UVATS) in various thoracic surgical procedures.^1^, ^2^, ^3^, ^4^ Despite more technical difficulties than UVATS, many surgeons are still eager to perform SVATS due to the advantages of being associated with lower postoperative pain and the ability to perform bilateral lesions and concomitant mediastinal and pulmonary lesions through a single incision.^5^, ^6^, ^7^ However, it is still debatable when weighing up the benefits claimed to be gained from a subxiphoid approach against its more technical difficulty, especially in some complex surgeries such as segmentectomy. Therefore, we conducted this study to evaluate the worthiness of SVATS segmentectomy through comparison of different perioperative variables, postoperative pain, quality of life and cost between SVATS segmentectomy and UVATS segmentectomy in patients with stage I non‐small cell lung cancer (NSCLC).

## Methods

From September 2014 to April 2018, we performed uniportal VATS segmentectomies in 687 patients. They were divided into two groups according to the approach used; 371 cases of UVATS segmentectomies and 316 cases of SVATS segmentectomies. From both groups, we included only patients who underwent ipsilateral single or multiple segmentectomies in one stage with proven stage IA lung cancer (with N0 status). We excluded cases who had undergone single stage bilateral lung resections, combined segmentectomy and lobectomy, combined segmentectomy and mediastinal lesion resection, previous ipsilateral pulmonary resection and cases where surgery was performed through hybrid uniportal and subxiphoid approaches. All patients were preoperatively evaluated and carefully selected by a multidisciplinary team to undergo SVATS segmentectomy. As selection criteria for patients undergoing SVATS, we excluded those with a body mass index (BMI) greater than 30 kg/m^2^ and patients with cardiac problems or arrhythmia.

After applying the aforementioned inclusion and exclusion criteria, our study comprised 278 patients in the UVATS group and 213 patients in the SVATS group. All cases were performed at the thoracic surgery department, Shanghai Pulmonary Hospital, Shanghai, China. Written informed consents were obtained from all patients for the surgical procedure and possible use of data in scientific research. The study was approved by the Institutional Review Board of the hospital (approval number: K17‐160).

All patients were subjected to routine preoperative clinical assessment, laboratory investigations, computed tomography (CT) scans of the chest, positron emission tomography scans if needed, pulmonary function tests and flexible bronchoscopy.

Demographic, clinical, operative and postoperative data were collected from patients' computerized records, medical notes, follow‐up files at the outpatient clinic (OPC), personal interviews and telephone calls. Postoperative pain scoring and quality of life questionnaire were applied to all patients. Hospital operative and postoperative costs were collected from the hospital's financial administration. All costs were analyzed and compared between both groups and expressed in Chinese renminbi (RMB).

### Surgical technique

**Figure 1:**
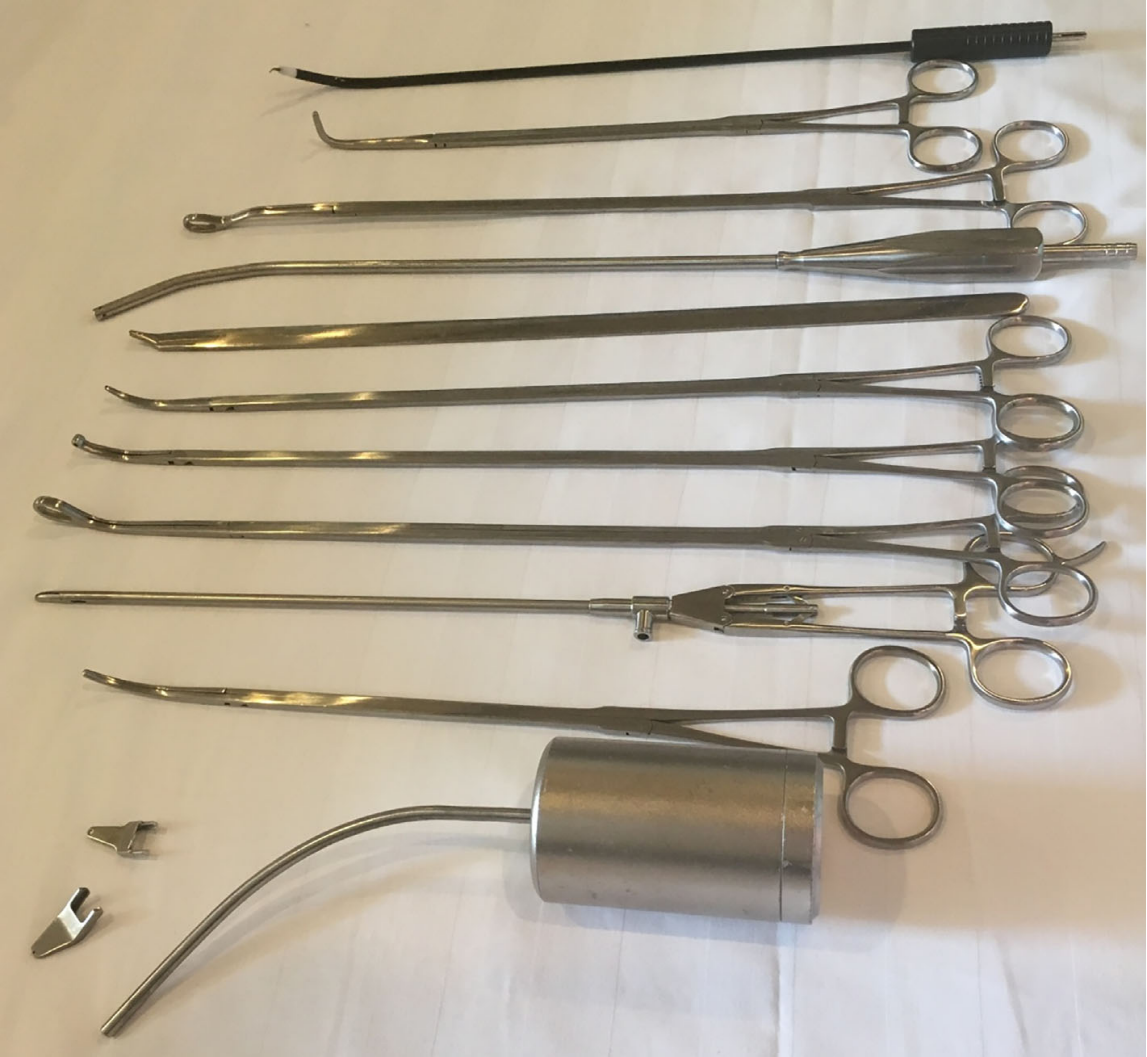
Specifically designed subxiphoid instruments (Shanghai Medical Instruments Group Ltd).

All surgical procedures on patients were performed under general anesthesia with double lumen tube (single lung ventilation). Only one monitor was used and placed above the head of the patient. The surgeon stood on the abdominal side of the patient while an assistant stood on the opposite side. A 10 mm, 30° angled videothoracoscope was used in all cases. The usual VATS instruments were used in UVATS cases, and specially designed longer instruments with more angled ends were used for SVATS segmentectomies (Shanghai Medical Instruments Group Ltd) (Fig 1).

**Figure 2:**
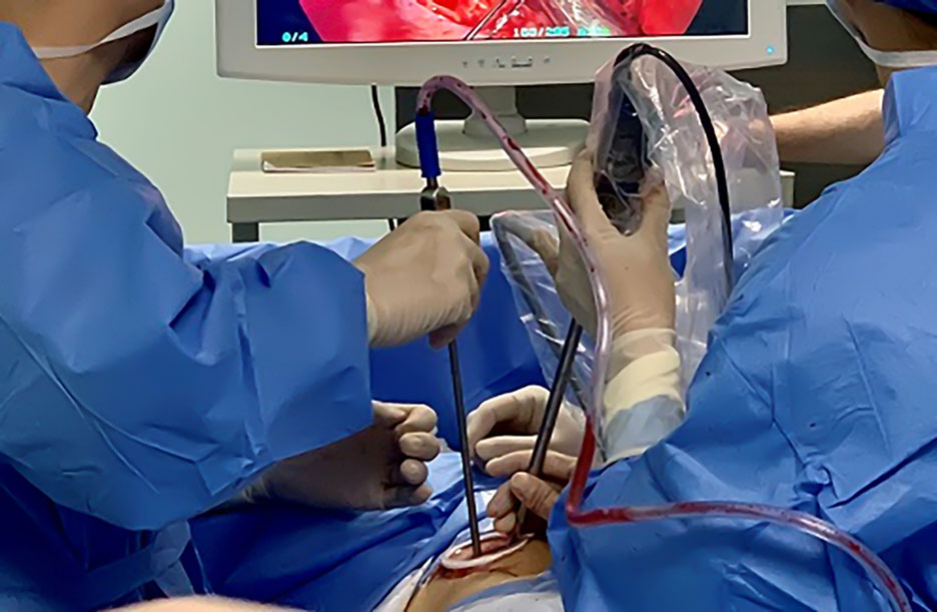
Intercostal incision between midaxillary and anterior axillary lines in the fourth or fifth intercostal space.

**Figure 3:**
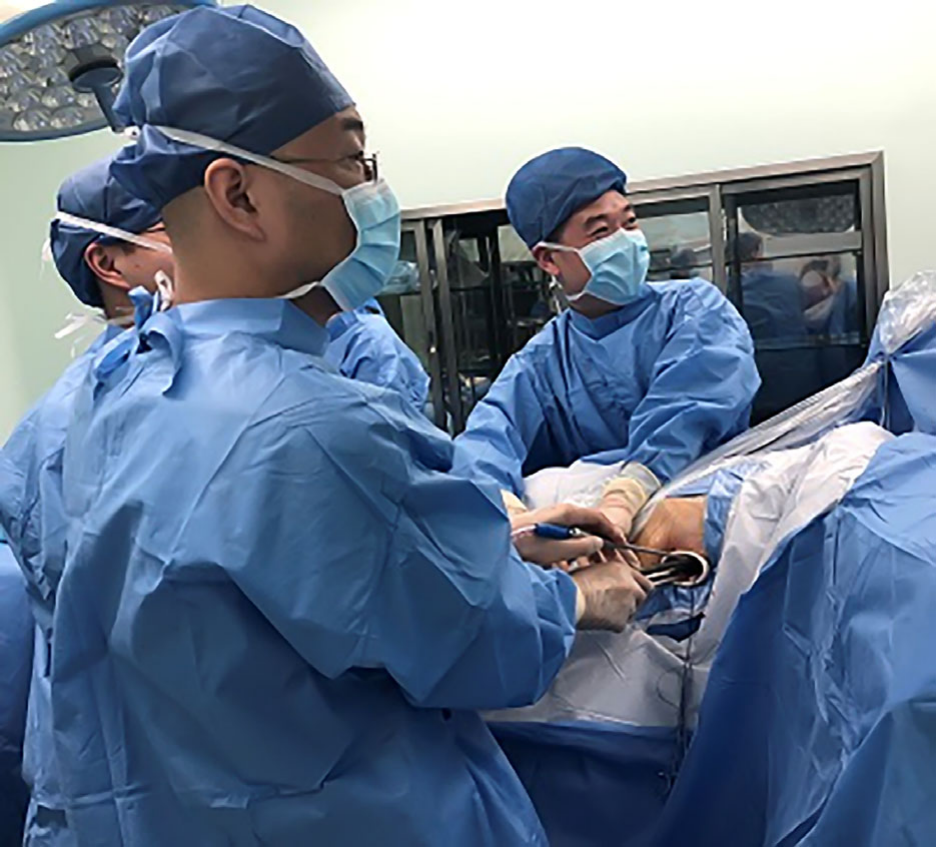
Subxiphoid incision extending from the xiphisternal junction to 1 cm below the xiphoid process.

Patients who were operated on via UVATS were positioned in lateral decubitus position and a 3–4 cm incision made between the midaxillary and anterior axillary lines in the fourth or fifth intercostal space according to the potentially resected segment (Fig 2). In case of SVATS, patients were placed in the lateral decubitus position with a slight backward inclination of 30° and a 4 cm longitudinal incision is made and extended from the xiphisternal junction to 1 cm below the xiphoid process (Fig 3). The operative field was sterilized widely to allow conversion to multiport, uniportal intercostal approaches or thoracotomy, as required.

### Surgical details

In case of UVATS, surgeons had direct access to the thoracic cavity. For the subxiphoid approach, the subcutaneous tissue was dissected, and then the rectus abdominis muscle was exposed and dissected longitudinally to expose the xiphoid process that was completely resected to provide a widened operative access. A retrosternal tunnel was created above the diaphragm by moving the index finger retrosternally, then more toward the operated side to bluntly dissect the pleura. A wound protector (Chinese Manufacture wound protector, Changzhou, China) was inserted to facilitate the passage of camera and instruments. The pleura of the operated side was then opened under thoracoscopic visualization and the pericardiophrenic fat was removed. Detailed surgical procedures of both approaches together with the intersegmental plan identification have been previously described in different publications.^4^, ^8^, ^9^, ^10^, ^11^

After segmental resection, the specimen was palpated to ensure the presence of the pulmonary lesion and adequacy of safety margin around it. The specimen was then sent routinely for frozen section diagnosis for primary pathological assessment. In case of negative palpation or nonadequate safety margin by inspection or frozen section results, an immediate completion lobectomy (CL) was performed. Systemic lymph node (LN) sampling was performed routinely from at least three N2 stations according to the IASLC/Mountain classification.

**Figure 4:**
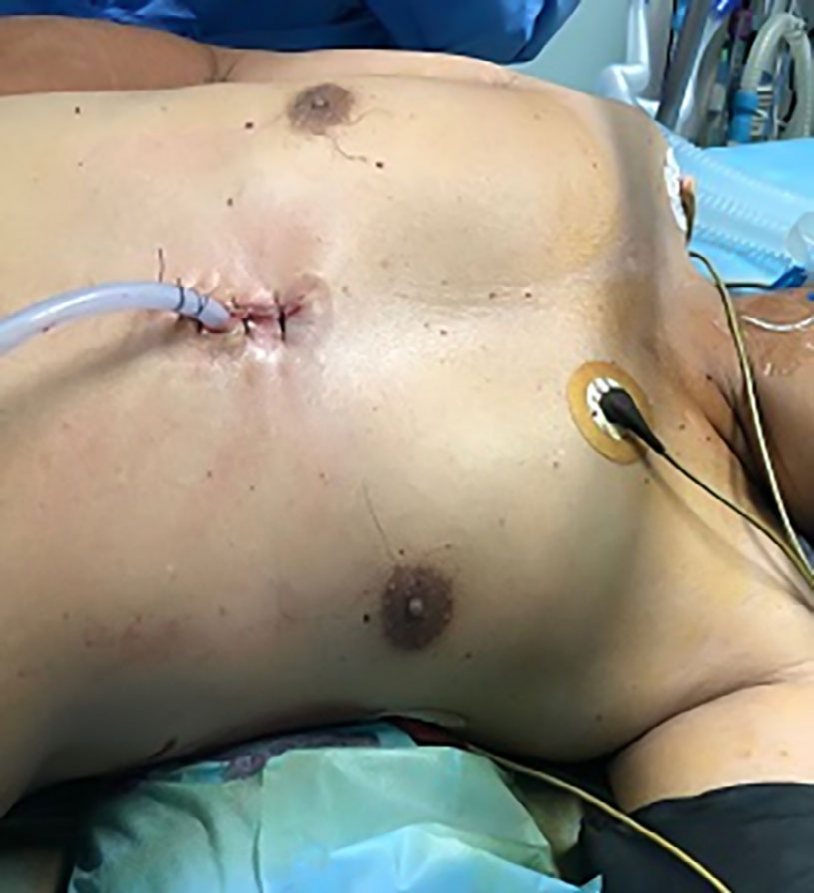
Subxiphoid incision with the drain at its lower end.

At the end of the surgery, a 28F pleural drain was inserted at the superior or inferior end of the incision in the intercostal approach. In the subxiphoid approach, the drain was inserted at the inferior end of the wound (Fig 4).

### Postoperative care of patients

All patients were extubated on the table then moved to the intensive care ward. They were then transferred to the normal ward if they were doing well with drainage of no more than 500 mL in the first four hours. The same protocol of postoperative pain control was followed in both groups by using a patient‐controlled analgesia pump with sufentanil citrate 1 mL: 50 μg. In addition, regular medications with flurbiprofen 50 mg every 12 hours, alternating with paracetamol 1 g every six hours were prescribed. Pain scoring was done for all patients using the visual analogue scale (VAS). The patient selected a number ranging from zero (no pain) to 10 (the most severe pain) that described his/her pain on a horizontal scale. Pain score was recorded after eight hours on postoperative day (POD) zero, POD1, POD3 and before discharge. The pleural drain was removed after any air‐leakage had ceased and with drainage less than 300 cc in 24 hours. Patients were usually discharged one day after the drain had been removed and seen two weeks, one month, three months, six months then every six months intervals postoperatively at the OPC.

Postoperative quality of life scoring was applied to our patients at OPC after three months, six months and one year intervals using the Chinese version of Quality of Life Questionnaire‐Lung Cancer 43 (EORTC QLQ‐LC43).^12^ Each question was scored from 1 to 4 according to how much the patient had experienced the subject of the question. A score of 1 was the minimum experience the patient had and a score of 4 indicated the maximum experience the patient had. The lower the overall score expressed, the better quality of life and vice versa. Postoperative follow‐up of our patients ranged from 13 months to 56 months.

### Statistical analysis

Our study was a nonrandomized retrospective comparative analysis. Data entry and statistical analysis were performed using SPSS (statistical package of social sciences) version 22 (SPSS Inc., Chicago, IL, USA). Parametric data were expressed in mean ± standard deviation. Non‐normal data were expressed as median, minimum and maximum. Normality of data was tested by one‐sample Kolmogorov‐Smirnov test. In addition, independent *t*‐test was used to compare means for continuous variables of each two different groups. Mann‐Whitney U test (z) was used to compare non‐normally distributed continuous variables in two different groups. Pearson Chi‐square tests were used to compare the categorical variables between groups. Fisher exact test was used when 50% of cell value was less than five. *P*‐value < 0.05 was considered as statistically significant.

## Results

Our study comprised 491 patients divided into 278 patients in the UVATS group and 213 patients in the SVATS group. Perioperative data are shown in (Table 1). The subxiphoid group showed a statistically significant higher percentage of forced expiratory volume in one second on the predicted value (FEV1%) (Table 1). That difference had no impact on the postoperative results. So, we did not need to further match patients' criteria. There was no significant difference between both groups regarding perioperative data size of the lesion on CT scan of the chest (Table 1).

**Table 1:** Patients' perioperative data

	Intercostal	Subxiphoid	*P*‐value
Age, mean ± SD	56.71 *±* 10.97	56.38 *±* 10.51	0.7
Gender, count (%)
Male	114 (41%)	70 (32.9%)	0.06
Females	164 (59%)	143 (67.1%)	
Smokers, count (%)	39 (14.1%)	28 (13.1%)	0.7
FEV1%, mean ± SD	89.58 *±* 16.40	93.86 *±* 15.27	0.003
BMI, mean ± SD	23.7 ± 0.7	23.7 ± 2.7	0.7
Lesion diameter on CT scan of chest (mm), mean ± SD	12.7 ± 5.3	12.4 ± 4.7	0.4
Operative duration (hours), mean ± SD	1.73 *±* 0.65	1.87 *±* 0.63	0.007
Intraoperative hemorrhage (mL), mean ± SD	63.81 *±* 44.96	85.12 *±* 109.6	0.004
Number of sampled LN stations, median (min‐max)	5 (3–7)	4 (2–8)	<0.001
Number of dissected LN, median (min‐max)	9 (4–16)	10 (3–19)	<0.001
Intercostal tube drainage (mL), mean ± SD	226.89 *±* 121.4	248.15 ± 156.5	0.2
Intercostal tube duration (days), mean ± SD	2.69 *±* 1.5	2.79 *±* 1.4	0.3
Postoperative hospital stay (days), Mean ± SD	3.7 *±* 1.9	3.8 *±* 1.7	0.3

BMI, body mass index; CT, computed tomography scan; FEV1, forced expiratory volume in one second; SD, standard deviation.

Operative time was significantly shorter and intraoperative bleeding was significantly lower in the UVATS group (Table 1).

Frequencies of left‐sided segmentectomies and posterior segmentectomies were significantly higher in the intercostal group (Table 2). Left S6 segmentectomy was the most frequent type of segmentectomy performed in the intercostal group (26 cases, 9.4%) while right S1 segmentectomy was the most frequent type of segmentectomy performed in the subxiphoid group (37 cases, 17.4%) (Table S1).

**Table 2:** Operated sides, frequency of posterior segmentectomies and pathological types

	Intercostal count (%)	Subxiphoid count (%)	Total count (%)	*P*‐value
Side				0.008
Right	128 (46.0%)	125 (58.7%)	253 (51.5%)	
Left	150 (54.0%)	88 (41.3%)	238 (48.5%)	
Number (%) of posterior segmentectomies	110 (39.6%)	48 (22.5%)	158 (32.2%)	<0.001
Pathological types				
Invasive adenocarcinoma	109 (39.2%)	75 (35.2%)	184 (37.5%)	
Microinvasive adenocarcinoma	71 (25.5%)	51 (23.9%)	122 (24.8%)	
Adenocarcinoma in situ	94 (33.8%)	86 (40.4%)	180 (36.5%)	
Squamous cell carcinoma	3 (1.1%)	1 (0.5%)	4 (0.8%)	
Large cell lung carcinoma	1 (0.4%)	0 (0.0%)	1 (0.2%)	

Number of sampled LN stations was significantly higher in the intercostal group, while the number of dissected LN from the sampled stations was significantly higher in the subxiphoid group (Table 1).

Invasive adenocarcinoma was the most frequent pathology in the intercostal group, while adenocarcinoma in situ was the most frequent in the subxiphoid group (Table 2).

Frequency of intraoperative arrhythmia was insignificantly higher in the subxiphoid group. There was no significant difference regarding rate of intraoperative conversion to thoracotomy or completion lobectomy after initial segmentectomy (Table 3).

**Table 3:** Operative and postoperative complications

Cause		Intercostal count (%)	Subxiphoid count (%)	Total count (%)	*P‐* value
Operative complications					
Operative arrhythmia		3 (1.1%)	7 (3.3%)	10 (2.0%)	0.08
Intraoperative conversion					
Completion lobectomy					0.2
Nonvisualized lesion/insufficient safety margin		5 (1.8%)	1 (0.5%)	6 (1.2%)	
Iatrogenic bronchial injury		1 (0.4%0	1 (0.5%)	2 (0.4%)	
Thoracotomy					
Bleeding		2 (0.7%)	2 (0.9%)	4 (0.8%)	
Arrhythmia		0 (0.0%)	1 (0.5%)	1 (0.2%)	
Uniportal intercostal					
Bleeding		0 (0.0%)	2 (0.9%)	2 (0.4%)	
Technical difficulty		0 (0.0%)	1 (0.5%)	1 (0.2%)	
Postoperative complications					
Prolonged air leak		7 (2.5)	4 (1.9)	11 (2.2%)	0.5
Arrhythmia		0 (0.0)%	1 (0.5%)	1 (0.2%)	
Re‐exploration for bleeding		1 (0.4%)	2 (0.9%)	3 (0.6%)	

Four patients in the intercostal group (1.4%) and three patients in the subxiphoid group (1.4%) underwent segmentectomy after a previous contralateral pulmonary wedge resection or segmentectomy. Neither 30 days hospital mortality nor one‐year same lobe locoregional recurrence was recorded in either group.

Postoperative pain score was statistically lower in the SVATS group on the POD zero, POD1, POD3 and before discharge. Postoperative quality of life score was statistically better in the SVATS group after three months, six months and one year assessment. UVATS segmentectomy appeared to be significantly cheaper than SVATS segmentectomy (Table 4).

**Table 4:** Postoperative pain scoring, quality of life, hospital cost

	Intercostal (mean ± SD)	Subxiphoid (mean ± SD)	*P*‐value
Postoperative pain scoring			
Postoperative pain (POD) zero	4.51 *±* 0.88	3.29 *±* 1.14	<0.001
Postoperative pain (POD) 1	4.25 *±* 0.61	2.68 *±* 0.80	<0.001
Postoperative pain (POD) 3	2.1 *±* 0.4	1.5 *±* 1.01	<0.001
Postoperative pain before discharge	1.8 *±* 0.36	0.94 *±* 0.7	<0.001
Quality of life score			
Quality of life after three months	68.10 *±* 2.55	66.49 *±* 2	<0.001
Quality of life after six months	64.86 *±* 2.21	63.17 *±* 1.53	<0.001
Quality of life after one year	60.95 *±* 1.36	60.22 *±* 0.71	<0.001
Cost (expressed in RMB)			
Cost, median (min‐max)	45 277 (35 967.69–66 711.48)	51 535 (34 535–61 100)	<0.001

POD, postoperative day; RMB, Chinese renminbi.

## Discussion

Our study included 278 patients in the UVATS group and 213 patients in the SVATS group. We selected patients with stage IA lung cancer to match as closely as possible the nature of the disease the patients were suffering from, in order to achieve a more uniform assessment of postoperative quality of life rather than assessment of some patients with malignant disease, and others with benign tumor or infectious disease.

Our selection criteria for any patient to undergo major lung resection was for the FEV1 to be >40%.^13^ In the current study, the subxiphoid group preoperatively showed a statistically significant higher FEV1%. However, that difference did not affect the postoperative clinical results of the intercostal group regarding suffering from respiratory issues, need for mechanical ventilation or prolonged hospital stay. Therefore, we did not need to further match patients' criteria regarding FEV1%.

Operative time was significantly shorter and intraoperative bleeding was significantly lower in the UVATS group, the results of which may coincide with the findings in the study of Cai *et al*.^6^ As known, UVATS segmentectomy started after a sufficient learning curve of other uniportal intercostal pulmonary procedures such as bullectomy and lobectomy.^14^, ^15^ The intercostal approach has also been considered primarily the default approach at our hospital. On the other hand, the subxiphoid approach, as with any new technique, took longer operative time especially in the early cases. Furthermore, the indirect access to thoracic cavity in SVATS takes more time than the UVATS with direct access to the thoracic cavity.

In both groups in our study, there was no significant difference regarding rate of intraoperative completion lobectomy, or conversion to thoracotomy. We experienced completion lobectomy after initial segmentectomy in eight cases (1.6%). The main reasons for this were nonvisualization of the lesion, or inadequate safety margin in six cases and severe iatrogenic injury to the lobar bronchus by the stapler in two cases, which emphasizes the importance of careful examination of the resected segment for presence of the lesion and ensuring that there is an adequate safety margin.

In total, major bleeding occurred on six occasions (1.2%); twice in the intercostal group and four times in the subxiphoid group. Two cases in the subxiphoid group were converted to an intercostal approach in order to control bleeding. The other four cases in both groups needed conversion to thoracotomy. Mild, but controllable, intraoperative bleeding was experienced in many other cases in both groups. However, control of bleeding through the subxiphoid approach was more difficult and took more time than the intercostal approach which has better direct access to vascular structures.^6^, ^11^ We therefore postulate the previous point as a leading cause for the significant increase of intraoperative blood loss in the subxiphoid group compared with the intercostal group.

Duration of chest tubes and postoperative hospital stays showed no significant difference between both groups, which is in agreement with the studies of Cai *et al*. and Song *et al*.^6^, ^13^

Intraoperative cardiac arrhythmia is known to occur during SVATS, especially in patients with left‐sided basal lesions due to a greater incidence of cardiac compression.^16^, ^17^, ^18^ One patient in the subxiphoid group experienced intraoperative ventricular fibrillation which necessitated urgent thoracotomy for resuscitation and cardiac message after a failed trial of direct current cardioversion (DCCV), but the situation was managed safely without any postoperative sequelae. Therefore, one of the precautions in preoperative case selection in SVATS is to avoid patients with a history of cardiac disease or arrhythmia, in particular those with left‐sided lesions.^4^ Backward tilting of the patient during positioning and use of longer and specially curved instruments with the concave edge of the instruments toward the pericardium during the operation have enabled surgeons to make better retraction with consequent lower compression on the heart.^11^

The number of posterior segmentectomies (S2, S6, S10 and left S [1+2] b) was significantly higher in the UVATS group as the access to posterior segments was more difficult through the subxiphoid approach in early cases. Also, one of our selection criteria during our initial subxiphoid experience was to avoid operating on patients with posterior lesions.^10^ However, with increasing experience and use of the specially designed long curved instruments, more challenging cases of posterior segmentectomies could be managed.^4^

Avoidance of intercostal nerve compression and decreased postoperative pain have been considered as fundamental advantages of subxiphoid approach.^5^, ^6^, ^13^, ^19^, ^20^ The postoperative pain score was statistically lower in the SVATS group on the POD zero, POD1, POD3 and before discharge. What is of particular note here is the decreased postoperative pain despite a longer operative time and proposed increased manipulation time through the operative incision. This may raise the question of whether we should consider a subxiphoid approach in surgeries with predicted prolonged operative time whenever feasible to avoid intense postoperative pain? The same findings were also reported by Cai *et al*.^6^, which indicated that there was decreased postoperative pain in spit of longer operative time in patients who underwent SVATS bilateral major lung resection compared to UVATS. It is hoped that future studies may handle this point with further details.

In order to further investigate if SVATS segmentectomy deserves going through its prolonged learning curve and more complex technique, we assessed and compared the overall postoperative quality of life score between UVATS and SVATS segmentectomy groups after three months, six months and one year using the Chinese version of Quality of Life Questionnaire‐Lung Cancer 43 (EORTC QLQ‐LC43).^12^ Postoperative quality of life has previously been reported in the literature with regard to the difference between VATS and open approaches^21^ as well as between multiport and uniportal approaches in early stage lung cancer.^22^ On one hand, the results came out in favor of the VATS approach over thoracotomy and on the other hand in favor of the uniportal over the multiport approach. According to our knowledge, we did not find previous studies which compared quality of life between SVATS and UVATS segmentectomy for early stage lung cancer.

In our study, postoperative quality of life overall score was statistically better (lower in numerical value) in the SVATS group after three months, six months and one year assessment. That might be a reflection from the better postoperative pain experienced by patients who were subsequently able to resume their normal activities and achieve a faster return to normal life.

Arguments about sufficient radicality and oncological effectiveness may arise in such primary results of newly practiced techniques such as SVATS segmentectomy.^23^ Regarding our study, the number of sampled LN stations was statistically higher in the intercostal group, while the number of the dissected LN from the sampled stations was statistically higher in the subxiphoid group. In fact, we experienced some difficulty in LN sampling at the beginning of our learning curve, in particular from the posteriorly located stations. However, with the increasing learning curve, we were able to perform a systematic LN sampling from at least three N2 stations (as per the IASLC/Mountain classification).^10^ Moreover, neither 30 days hospital mortality nor one‐year same lobe locoregional recurrence was recorded in either groups. However, such outcomes were not the primary endpoints in our study, hence further detailed studies are needed to investigate long‐term survival and oncological efficacy of SVATS segmentectomy.

SVATS segmentectomy appeared to be significantly more expensive than UVATS segmentectomy which may be attributed to the increased cost of the specially designed instruments for SVATS compared with the usual uniportal intercostal instruments. Furthermore, the axis for SVATS segmentectomy necessitated use of the more expensive articulating staplers or reloads more frequently than in the intercostal approach. Moreover, in UVATS, in order to lever up the segmental blood vessels before passage of the stapler, a silk suture was passed around the blood vessel and then the suture was pulled up towards the laterally placed intercostal incision. However, in SVATS, the axis is different and pulling of the levering silk suture around the segmental blood vessel is directed mostly downwards and not laterally with minimal or no levering effect. Therefore, we used the curved tip vascular reloads more frequently as a levering tool to overcome that issue. Curved tip vascular reloads are more expensive than the ordinary vascular reloads. From the aforementioned postulations, we can see that most of the cost is due to operative supplies. However, we should not neglect the other side of the equation which is represented by better postoperative pain and better quality of life with supposed better productivity reflecting on the performance of the patients, family members and the society. Therefore, further in‐depth studies on the overall cost effectiveness of SVATS segmentectomy should be conducted in the future.

In our opinion, some preoperative selection criteria of patients in the subxiphoid group and nonrandomization may have introduced some sort of selection bias. Also, the follow‐up period of 13–56 months was insufficient to assess long‐term survival and oncological efficacy.

To conclude, in spite of its technical difficulty, SVATS segmentectomy for stage IA lung cancer is a safe and feasible approach that is worth proceeding with as it is associated with better postoperative pain and better quality of life in the first postoperative year. SVATS segmentectomy is expensive but associated with a better quality of life. Therefore, further detailed studies should be conducted to evaluate its real cost‐effectiveness. Further randomized studies with longer follow‐up periods with particular attention to patient survival are recommended.

## Disclosure

The authors have no conflicts of interest to declare.

## Supporting information


**Table S1.** Types of segmentectomies.
