# Supplementary material for: Subxiphoid uniportal thoracoscopic pulmonary segmentectomy for stage I non‐small cell lung cancer: Feasibility, quality of life and financial worthiness
Source: Thorac Cancer. 2020 Mar 28;11(6):1414–22. doi: 10.1111/1759-7714.13392 (PMC7262898; doi:10.1111/1759-7714.13392)
Supplement: Supplementary file 1 — Table S1. Types of segmentectomies. [file TCA-11-1414-s001.docx]

Supplementary table 1: Types of segmentectomies

|  | Intercostal  *Count (%)* | Subxiphoid  *Count (%)* | Total  *Count (%)* |
| --- | --- | --- | --- |
| Right S1 | 22(7.9%) | 37(17.4%) | 59(12%) |
| Right S2 | 23(8.3%) | 29(13.6%) | 52(10.6%) |
| Right S3 | 16(5.8%) | 14(6.6%) | 30(6.1%) |
| Right S1+S2 | 8(2.9%) | 2(9%) | 10(2.0%) |
| Right S1+S3 | 8(2.9%) | 1(5%) | 9(1.8%) |
| Right S2+S3 | 1(4%) | 0(0%) | 1(.2%) |
| Right S4 | 2(.7%) | 10(4.7%) | 12(2.4%) |
| Right S5 | 3(1.1%) | 3(1.4%) | 6(1.2%) |
| Right S6 | 20(7.2%) | 8(3.8%) | 28(5.7%) |
| Right S8 | 3(1.1%) | 5(2.3%) | 8(1.6%) |
| Right S9 | 2(.7%) | 0(0%) | 2(.4%) |
| Right S10 | 5(1.8%) | 1(5%) | 6(1.2%) |
| Right lower basal segment | 5(1.8%) | 7(3.3%) | 12(2.4%) |
| Right S9+S10 | 1(4%) | 0(.0%) | 1(2%) |
| Right S8+S9 | 0(.0%) | 2(.9%) | 2(.4%) |
| Right S4+S6 | 0(.0%) | 3(1.4%) | 3(.6%) |
| Right S1+ Right lower basal segment | 0(.0%) | 1(5%) | 1(.2%) |
| Right S1+S6 | 3(1.1%) | 0(.0%) | 3(.6%) |
| Right S1+S4 | 1(4%) | 0(.0%) | 1(.2%) |
| Right S2+S6 | 5(1.8%) | 0(.0%) | 5(1.0%) |
| Right S3+S6 | 0(0%) | 1(.5%) | 1(2%) |
| Right S6+S10 | 0(.0%) | 1(5%) | 1(.2%) |
| Right S6+S8 | 0(.0%) | 1(.5%) | 1(.2%) |
| Right S3+S6+S8 | 1(4%) | 0(0%) | 1(2%) |
| Left (S1-2) a | 14(5.0%) | 20(9.4%) | 34(6.9%) |
| Left (S1-2) b | 18(6.5%) | 4(1.9%) | 22(4.5%) |
| Left S3 | 10(3.6%) | 4(1.9%) | 14(2.9%) |
| Left S6 | 26(9.4%) | 3(1.4%) | 29(5.9%) |
| Left S8 | 5(1.8%) | 4(1.9%) | 9(1.8%) |
| Left S9 | 1(.4%) | 0(0%) | 1(2%) |
| Left S10 | 7(2.5%) | 1(.5%) | 8(1.6%) |
| Left S1+S2 (S1-2) a & b | 5(1.8%) | 4(1.9%) | 9(1.8%) |
| Left (S1-2) a+S3 | 3(1.1%) | 2(.9%) | 5(1.0%) |
| Left (S1-2) b+S3 | 5(1.8%) | 0(.0%) | 5(1.0%) |
| Left upper trisegmentectomy ((S1-2) +S3) | 21(7.6%) | 27(12.7%) | 48(9.8%) |
| Left upper lingulectomy | 19(6.8%) | 7(3.3%) | 26(5.3%) |
| Left lower basal segment | 9(3.2%) | 8(3.8%) | 17(3.5%) |
| Left S3+ lingulectomy | 2(7%) | 0(.0%) | 2(.4%) |
| Left (S1-2) b+S6 | 1(.4%) | 1(5%) | 2(.4%) |
| Left S6+ left upper trisegmentectomy | 0(.0%) | 1(5%) | 1(.2%) |
| Left S8+S10 | 0(0%) | 1(.5%) | 1(2%) |
| Left S9+S10 | 1(4%) | 0(0%) | 1(2%) |
| Left S3+S6 | 1(4%) | 0(.0%) | 1(.2%) |
| Left (S1-2) b+S3 | 1(.4%) | 0(.0%) | 1(.2%) |
